# NiN-Passivated NiO Hole-Transport Layer Improves Halide Perovskite-Based Solar Cell

**DOI:** 10.1021/acsami.2c11701

**Published:** 2022-10-13

**Authors:** Anat Itzhak, Xu He, Adi Kama, Sujit Kumar, Michal Ejgenberg, Antoine Kahn, David Cahen

**Affiliations:** † Department of Chemistry and Bar-Ilan Institute for Nanotechnology & Advanced Materials, Bar-Ilan University, Ramat Gan5290002, Israel; ‡ Department of Electrical and Computer Engineering, Princeton University, Princeton, New Jersey08544, United States; § Weizmann Institute of Science, Rehovot7610001, Israel

**Keywords:** halide perovskites, solar cells, nickel oxide, nickel nitride, passivation, interface

## Abstract

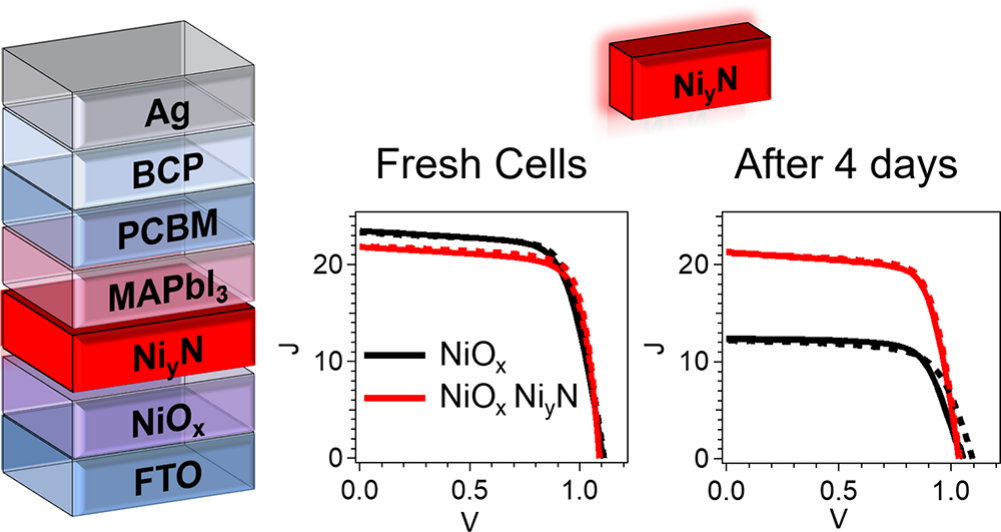

The interfaces between inorganic selective contacts and halide perovskites (HaPs) are possibly the greatest challenge for making stable and reproducible solar cells with these materials. NiO_*x*_, an attractive hole-transport layer as it fits the electronic structure of HaPs, is highly stable and can be produced at a low cost. Furthermore, NiO_*x*_ can be fabricated via scalable and controlled physical deposition methods such as RF sputtering to facilitate the quest for scalable, solvent-free, vacuum-deposited HaP-based solar cells (PSCs). However, the interface between NiO_*x*_ and HaPs is still not well-controlled, which leads at times to a lack of stability and *V*_oc_ losses. Here, we use RF sputtering to fabricate NiO_*x*_ and then cover it with a Ni_*y*_N layer without breaking vacuum. The Ni_*y*_N layer protects NiO_*x*_ doubly during PSC production. Firstly, the Ni_*y*_N layer protects NiO_*x*_ from Ni^3+^ species being reduced to Ni^2+^ by Ar plasma, thus maintaining NiO_*x*_ conductivity. Secondly, it passivates the interface between NiO_*x*_ and the HaPs, retaining PSC stability over time. This double effect improves PSC efficiency from an average of 16.5% with a 17.4% record cell to a 19% average with a 19.8% record cell and increases the device stability.

## Introduction

Halide perovskite solar cells (PSCs) have been extensively researched since 2009, and their power conversion efficiency (PCE) has improved toward their Shockley–Queisser limit. One of the main factors for the high PCE in PSCs composed of polycrystalline thin films is the intrinsically low defect concentration in the bulk of halide perovskites (HaPs).^1−3^ However, PSCs are not constructed from HaPs alone, and defect states at interfaces between HaP films and adjacent layers adversely affect the conversion efficiency, long-term stability, and reproducibility of PSCs.^4,5^

Nickel oxide (NiO_*x*_) is a sturdy and efficient hole-transport material (HTM), which has been reported to improve the stability of PSCs over that achieved with organic HTMs.^6−9^ Radio-frequency (RF) sputtering is a scalable fabrication method for metal oxides with a highly controlled oxygen partial pressure. Wang et al. have shown that sputtered NiO_*x*_ at low temperatures has controlled transparency and conductivity that affect the efficiency of the PSC.^10^ Moreover, sputtered NiO_*x*_ is preferred over wet-chemically processed NiO_*x*_ because it leaves no residues of solvents or precursors that can damage the stability and efficiency of the final device.^11^ However, sputtered NiO_*x*_ hole-transport layers (HTLs) with no further treatment have parasitic resistance that leads to PSCs with moderate fill factors (FFs) and efficiencies.^12−14^

One of the challenges of using NiO_*x*_ as HTM is its nonstoichiometric composition; Ni^2+^ readily oxidizes into Ni^3+^. Then, charge balance leads to a Ni-poor material, NiO_*x*_ with, i.e., *x* < 1, which is a p-type semiconductor due to Ni vacancies. Excess oxygen in more conductive NiO_*x*_ leads to more Ni^3+^ species.^15^ On the one hand, the Ni^3+^ cations are essential as dopants to improve NiO_*x*_ conductivity.^16,17^ On the other hand, the same Ni^3+^ cation is highly reactive and leads to degradation of the adjacent HaP layer in the solar device.^14^

Earlier attempts to passivate the NiO_*x*_/HaP interface have improved PSC efficiency and stability.^18−20^ Most passivation techniques have so far included deposition of a buffer layer through spin coating^21,22^ or introducing additives into the HaP solution.^23^ These passivation approaches are not suited for the fabrication of larger area perovskite devices that would inevitably need solvent-less methods, for both active layer deposition and interface modification.^24,25^

In this work, we use RF magnetron sputtering for an alternative in-situ route to passivate the HaP/NiO_*x*_ interface. The approach is well-suited for upscaling PSC throughput via all vacuum-processed device fabrication techniques. We deposit nickel nitride (Ni_*y*_N), a very small bandgap semiconductor, as an ultra-thin ∼2 nm layer on NiO_*x*_, essentially an in-situ modification of the NiO_*x*_ surface without breaking vacuum. This Ni_*y*_N interlayer becomes a buffer between the oxide and the HaP film. We then investigate its effects on PSC performance and stability.

We find that Ni_*y*_N protects NiO_*x*_ from the reduction of Ni^3+^ cations to Ni^2+^ during the Ar plasma cleaning step, typically used to improve the wettability of the oxide, thus maintaining NiO_*x*_ conductivity. The Ni_*y*_N layer also passivates the interface between NiO_*x*_ and MAPbI_3_ and inhibits the reaction between MAPbI_3_ and Ni^3+^. Although Ni_*y*_N is conductive and could be expected to introduce trap states that damage PV performance, we show that thin enough Ni_*y*_N can passivate the interface between NiO_*x*_ and HaP, thereby improving device efficiency and stability.

## Experimental Section

### Fabrication

#### Substrate Cleaning

Fluorine-doped tin oxide (FTO, KINTEC Company)-coated glass substrates (TEC 15, 1 inch × 1 inch) were cleaned in a sonication bath with soap (Decon 90) and deionized water and then rinsed in deionized water followed by dry ethanol.

NiO_*x*_ and Ni_*y*_N were deposited using RF sputtering (AJA International Inc.) from 2-inch NiO and Ni targets, respectively (Kurt J. Lesker, 99.9%). NiO_*x*_ deposition was done at room temperature with an Ar gas flow of 30 sccm and a total chamber pressure of 3 mTorr. Ni_*y*_N deposition was done at room temperature and a total chamber pressure of 3 mTorr, with Ar and N_2_ gas flows of 15 and 45 sccm, respectively. In the first stage, the NiO_*x*_ target was set to 80 W for 1 h, then the chamber was purged for 10 min, and finally, the Ni target was set to 60 W for 3 min. Ar plasma cleaning was performed by applying a power of 30 W on the substrate with an Ar flow of 30 sccm.

MAPbI_3_ was synthesized from MAI (Greatcell Solar) and PbI_2_ (Sigma-Aldrich, 99.999%) precursors in a 1:1 ratio at a concentration of 1.5 M. The precursors were dissolved overnight at 60 °C in γ-butyrolactone (GBL, Alfa Aesar, 99%) and dimethyl sulfoxide (DMSO, Sigma-Aldrich, anhydrous) at a 7:3 ratio. The solution was spin-coated at 4000 rpm for 35 s with 800 μL of a toluene (Sigma-Aldrich, anhydrous) anti-solvent drip after 30 s. For device fabrication, 20 mg of PCBM was dissolved in 1 mL of chlorobenzene overnight, spin-coated at 2000 rpm for 30 s, and annealed for 10 min. After 15 min of cooling, a solution of 3 mg of bathocuproine in 6 mL of isopropanol was spin-coated at 4000 rpm for 30 s. Finally, thermal evaporation was performed to deposit round Ag contacts of 3 mm diameter. A scheme of the device structure is given in the Supporting Information (SI) (Figure S1).

### Characterization

Ultraviolet photoemission spectroscopy (UPS) was used to probe the vacuum level and the position of the Fermi level with respect to the valence band edge, leading to the determination of the work function (WF) and ionization energy (IE) of the measured materials at a resolution of 0.15 eV. UPS was performed in an ultrahigh vacuum (10^–10^ Torr) with He I photons (21.22 eV) generated by a He discharge lamp, with a pass energy of 5 eV and a 0.02 eV step size.

X-ray photoelectron spectroscopy (XPS) with an Al Kα anode (1486.6 eV) was used to probe the Ni 2p, N 1s, and C 1s core levels at a resolution of 0.8 eV. Scans were taken with a pass energy of 25 eV and a 0.05 eV step size at a low base pressure of 10^–9^ Torr. UPS and XPS were conducted on the samples before and after a 5 s Ar^+^ etching. The Ar^+^ etching was performed using an Ar ion gun at a pressure of 5.5 × 10^–5^ Torr, a 1000 V acceleration voltage, and a 20 mA emission current. The current measured during the etching process was around 15 μA. All UPS, XPS, and Ar^+^ etching steps were performed in the same vacuum chamber without sample exposure to ambient atmosphere.

XPS in Bar-Ilan University (BIU) was performed using a Thermo Scientific Nexsa spectrometer XPS system with an Al Kα anode (1486.6 eV) at a base pressure of ∼7 × 10^–8^ Pa (∼5 × 10^–10^ Torr). The binding energy (BE) was calibrated vs carbon (C 1s = 285 eV). Survey scans were collected with a pass energy of 200 eV and a 1.0 eV step size, followed by high-resolution scans with a pass energy of 50 eV and a step size of 0.1 eV. The samples were exposed for ∼1 min to air during the sample transfer.

Optical transmission measurements were performed using an optical fiber-based custom-made system that consists of a CCD array spectrometer (USB4000, Ocean Optics) and two integrating spheres. The measurements were done over a 400–1000 nm spectral range with a circular diameter of 3 mm in a N_2_ atmosphere.

Two-probe measurements were done with a Keithley 2400 source measurement unit (SMU) in a potential range of −0.5 to 0.5 V at a 50 mV/point rate.

*J*–*V* characteristics were measured with the same Keithley source at a potential range of −1.2 to 0.2 V at a 20 mV/point rate in ascending and descending scan directions. The device was illuminated through an optical fiber using a laser-driven light source (LDLS, EQ-99FC, Energetiq) xenon lamp calibrated to the AM1.5G solar spectrum.

Photoemission yield spectroscopy (PEYS) was used to measure the IE (valence band maximum energy, relative to the vacuum level) of perovskite surfaces. The measurements were done under a N_2_ atmosphere, using an air photoemission system (ASKP150200, KP Technology Ltd.), illuminated by a deuterium (D_2_) UV source, coupled with a motorized grating monochromator.

Transient PL decay measurements on the HaP thin films deposited on NiO/FTO substrates with and without a NiN interlayer were measured in ambient conditions. The HaP thin films were excited with a 450 nm picosecond pulsed laser diode source, and PL decay characteristics in the 760–765 nm emission wavelength range were recorded with a photomultiplier tube.

## Results and Discussion

We used RF sputtering to deposit a NiO_*x*_ layer from a NiO target on an FTO-coated glass substrate. To modify the NiO_*x*_ surface, we used reactive sputtering treatment of the Ni target with a plasma composition of 25% Ar and 75% N_2_.

**Figure 1:**
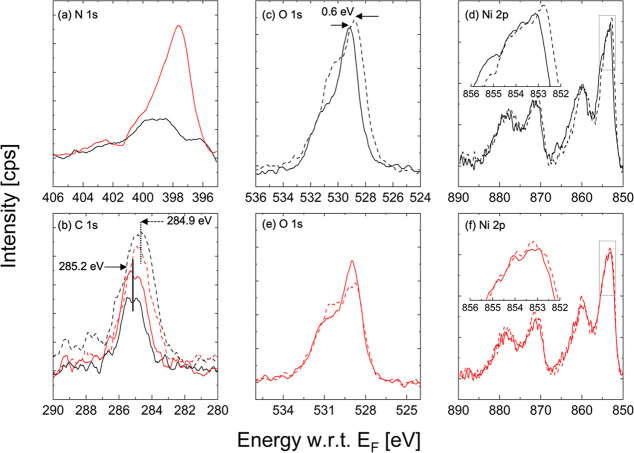
Narrow-range XPS plots of the (a) N 1s peak of a reference NiO_*x*_ (black) and Ni_*y*_N-modified NiO_*x*_ (red), (b) C 1s peak of a reference NiO_*x*_ (black) and Ni_*y*_N-modified NiO_*x*_ (red) before (dashed) and after (solid) Ar^+^ etching, (c) O 1s and (d) Ni 2p peaks of a reference NiO_*x*_ before (dashed) and after (solid) Ar^+^ etching, and (e) O 1s and (f) Ni 2p peaks of a Ni_*y*_N-modified NiO_*x*_ before (dashed) and after (solid) Ar^+^ etching.

To examine the reactive sputtering effect on the NiO_*x*_ surface, we used XPS on NiO_*x*_ samples modified and unmodified by reactive sputtering. The XPS spectra from both NiO_*x*_ samples show a broad and shallow nitrogen signal around a binding energy of 399 eV, which typically fits the signal of nitrogen in organic matrices (Figure 1a).^26,27^ However, the NiO_*x*_ layer after a reactive sputtering treatment of a Ni target with a plasma composition of 25% Ar and 75% N_2_ exhibits a clear, sharp nitrogen peak at a binding energy of 397.7 eV that fits metal nitrides.

To examine the effect of the Ni_*y*_N modification on NiO_*x*_, we conducted 5 s of Ar^+^ etching to clean the surface of contaminants and mimic the layers built into the solar cells. After Ar^+^ etching, the intensity of C 1s is reduced, indicative of the surface cleaning effect of the 5 s etching process. The BE of the C 1s core level increases by 0.3 eV, from 284.9 to 285.2 eV, for both unmodified NiO_*x*_ and Ni_*y*_N-modified NiO_*x*_. Any carbon present in the layers is adventitious, and the BE difference is most likely due to a change in the chemical environment between the top and sub-surface species, as the surface is Ar^+^ etched (Figure 1b).

Nickel and oxygen are not adventitious; hence, their BE can be more directly linked to the Fermi level (*E*_F_) position in the material. The O 1s feature is a superposition of two core level peaks corresponding to oxygen bound to Ni^3+^ and Ni^2+^. The BE of the O 1s peak is found to increase by 0.6 eV upon Ar^+^ etching, from 528.6 to 529.2 eV, for NiO_*x*_ (Figure 1c) but only by 0.2 eV, from 528.7 to 528.9 eV, for Ni_*y*_N-modified NiO_*x*_ (Figure 1e). O 1s peak fitting details can be found in Figure S2. Similarly, the BE of the tallest Ni 2p peak increases by 0.4 eV from 852.7 to 853.1 eV for NiO_*x*_ (Figure 1d) but remains unchanged at 853.2 eV for Ni_*y*_N-modified NiO_*x*_ (Figure 1f).

**Figure 2:**
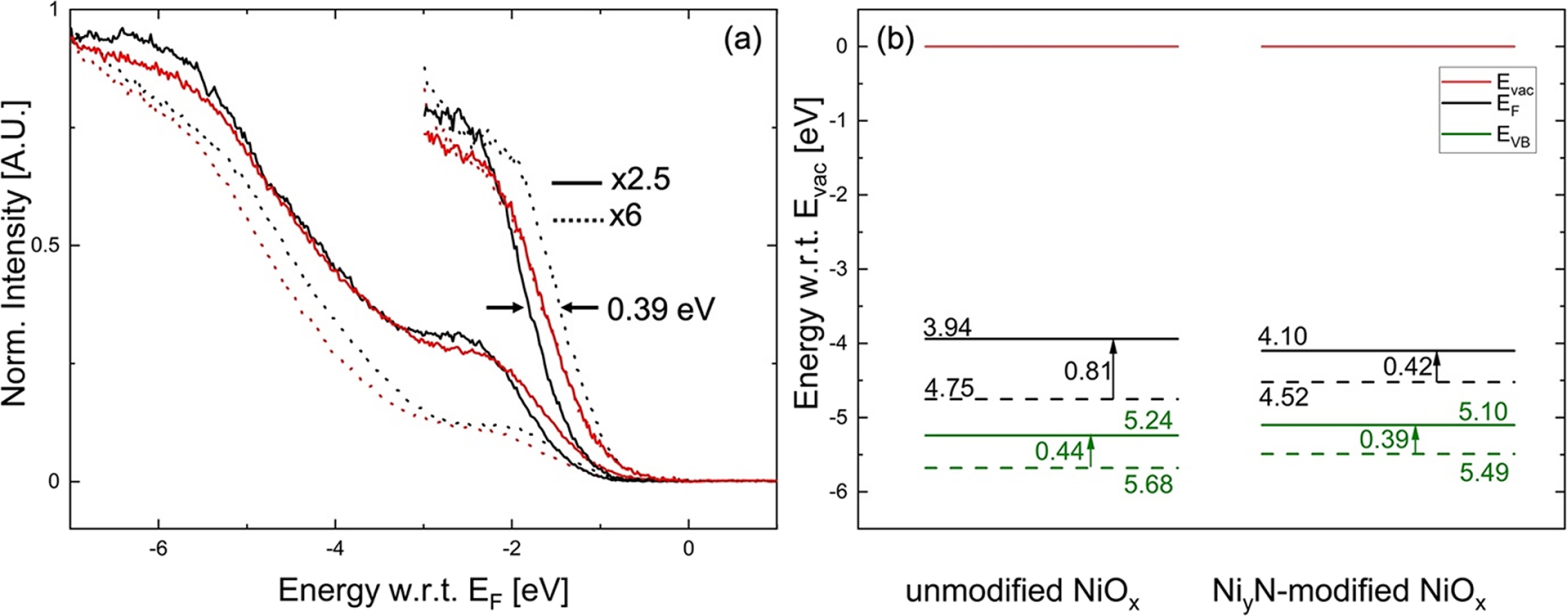
(a) Normalized UPS valence spectra plotted with respect to the Fermi level (*E*_F_) for NiO_*x*_ (black) and Ni_*y*_N-modified NiO_*x*_ (red) before (dashed) and after (solid) 5 s Ar^+^ etching. (b) Energy diagrams for NiO_*x*_ (left) and Ni_*y*_N-modified NiO_*x*_ (right) before (dashed) and after (solid) 5 s Ar^+^ etching. All energy levels are plotted with respect to the vacuum level (*E*_vac_). Energy diagrams plotted relative to the Fermi energy (*E*_f_) are shown in the SI (Figure S4).

To understand the effect of Ar^+^ etching on the electronic structure of the NiO_*x*_ surface, we performed UPS to determine the WF and IE of the NiO_*x*_ films, both with and without Ni_*y*_N modification. The valence band maximum (VBM) positions obtained by linear extrapolation of the leading edge of the filled states are shown in Figure 2a. Notably, the NiO_*x*_ valence band shows a 0.39 eV shift away from *E*_F_ upon Ar^+^ etching, whereas the Ni_*y*_N-modified NiO_*x*_ valence band remains at the same position (negligible shift of 0.03 eV). Comparing the valence bands of the NiO_*x*_ reference and Ni_*y*_N-modified NiO_*x*_ after 5 s of Ar^+^ etching, we find the VBM to be 0.30 eV closer to the Fermi level for Ni_*y*_N-modified NiO_*x*_ (1.00 eV below *E*_F_) than for the unmodified NiO_*x*_ (1.30 eV below *E*_F_) (Figure 2). This trend is further investigated by measuring a 40 nm thick Ni_*y*_N on NiO_*x*_, whose VBM reaches all the way to the Fermi level, forming almost a Fermi step (Figure S3).

The resulting energy diagrams for NiO_*x*_ (left) and Ni_*y*_N-modified NiO_*x*_ (right) before and after 5 s Ar^+^ etching, plotted relative to the vacuum level (*E*_vac_), are shown in Figure 2b. For clarity, similar energy diagrams, plotted relative to the Fermi energy (*E*_f_), as they were measured, are shown in the SI (Figure S4). The WF for unmodified NiO_*x*_ decreases from 4.75 to 3.94 eV after 5 s Ar^+^ etching and from 4.52 to 4.10 eV for Ni_*y*_N-modified NiO_*x*_. Together with the linearly extrapolated VBM values, the IE values are found to be 5.24 eV for Ar^+^-etched, unmodified NiO_*x*_ and 5.10 eV for Ni_*y*_N-modified NiO_*x*_ (Figure 2b). The UPS and XPS data suggest that the Ni_*y*_N-modified NiO_*x*_ is more resistant to damage or to defects induced by the Ar^+^ etching process than the unmodified NiO_*x*_. The BEs of the Ni 2p and O 1s core levels remain comparable before and after Ar^+^ etching for Ni_*y*_N-modified NiO_*x*_ within the resolution of XPS, indicative of a nearly unchanged Fermi level position before and after the etching process. At the same time, the unmodified NiO_*x*_ layer shows an increase in BE for both Ni 2p and O 1s core levels, reflecting a Fermi level shift toward the middle of the NiO_*x*_ gap.

NiO_*x*_ is typically not fully stoichiometric. Based on the presence of Ni^3+^ (Figure S2) and the p-type character of the film, as shown by the position of the Fermi level in the lower part of the gap (Figure 2b), we infer that the NiO_*x*_ we synthesized is O-rich as reported in the literature.^10,28^ The charge carrier (hole) density in the NiO_*x*_ is the result of nickel vacancies (V_Ni_^″^) and/or oxygen interstitials (O_i_^″^).^29^ Ar^+^ etching presumably creates more oxygen vacancies (V_O_^··^), because oxygen is lighter than nickel and, thus, more easily ejected from the etched surface. Oxygen vacancies can reduce the charge carrier concentration as , and thereby reduce the p-doping level of the NiO_*x*_ films. Ni_*y*_N-modified NiO_*x*_ is more resistant to such a process, as seen by the XPS and UPS results, demonstrating the passivating effects of Ni_*y*_N on the NiO_*x*_ surface. This passivating effect makes the Ni_*y*_N-modified NiO_*x*_ film less prone to electronic structure changes during subsequent processing steps, which is further corroborated by the unchanged energies of the valence states with respect to the Fermi level in Figure 2a.

As a minor point, the decrease in the adventitious C 1s peak intensity upon Ar^+^ etching of both surfaces (Figure 1b) is consistent with a ∼0.4 eV vacuum level (*E*_vac_) shift, which agrees with the 0.42 eV change in the Ni_*y*_N-modified NiO_*x*_ WF measured by UPS (a decrease in *E*_vac_ from 4.52 to 4.10 eV as seen in Figure S4) above *E*_F_ upon Ar^+^ etching (Figure 2b). For unmodified NiO_*x*_, however, the WF decreases by 0.8 eV, a combination of an ∼0.4 eV decrease in *E*_vac_ and an ∼0.4 eV downward shift of the band edges with respect to *E*_F_. To check if the effect of Ar^+^ etching performed in the XPS measurements resembles that of the Ar plasma cleaning, performed via RF sputtering, of the unmodified NiO_*x*_ and Ni_*y*_N-modified NiO_*x*_ surfaces, we used 60 s RF sputtering with Ar plasma to etch NiO_*x*_ with and without Ni_*y*_N modification, to imitate the conditions before MAPbI_3_ spin coating.

**Figure 3:**
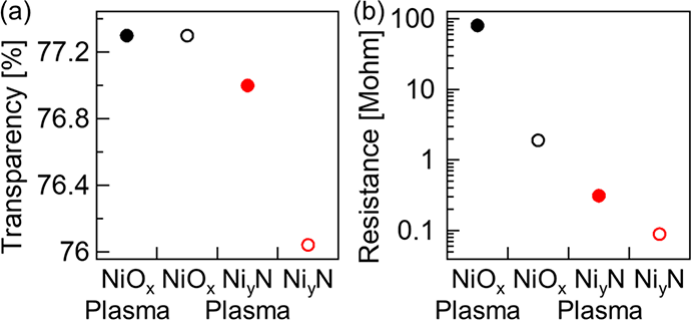
(a) UV–vis total optical transmission (TT) and (b) electrical resistance (logarithmic scale) measurement data of a reference NiO_*x*_ (black) and of a Ni_*y*_N-modified NiO_*x*_ (red) sample that was not etched (open) and after Ar plasma etching for 60 s (solid). Each mark in both plots is the average of nine measured points with an error range of ±0.4% for the transparency and ±10% for the resistance measurements.

The impact of the Ar plasma on the optical transparency of the unmodified NiO_*x*_ and the Ni_*y*_N-modified NiO_*x*_ was examined via total transmission measurements over a range of 370–950 nm. We found that in the visible range, Ar plasma treatment did not change the transparency of the unmodified NiO_*x*_. The Ni_*y*_N modification reduced NiO_*x*_ transparency by ∼1.5%, which is expected because of the narrow band gap of Ni_*y*_N. The Ar plasma treatment etched away part of the Ni_*y*_N and improved the transparency to bring it closer to that of the unmodified NiO_*x*_ (Figure 3a). The Ar plasma effect on the Ni_*y*_N-modified NiO_*x*_, together with the lack of change in the *E*_F_ position in the material’s gap seen by XPS and UPS, suggests that the Ni_*y*_N forms a protective layer on top of the NiO_*x*_, preventing a reaction between the NiO_*x*_ and the Ar plasma.

The NiO_*x*_ film protection by the Ni_*y*_N layer from the Ar plasma was investigated via two-probe electrical measurements, inspecting the resistance along the *Z* axis (perpendicular to the surface) between the FTO electrode and the NiO_*x*_ surface with and without a Ni_*y*_N layer, both before and after Ar plasma treatment. The Ar plasma treatment dramatically increased the unmodified NiO_*x*_ resistance ∼40×, from 1.9 MΩ before to 80 MΩ after. The Ni_*y*_N layer reduced the NiO_*x*_ resistance from 1.9 to 0.09 MΩ before plasma treatment, but even more critically, after Ar plasma treatment, the resistance increased about three times to 0.3 MΩ, lower than that of the unmodified NiO_*x*_ (Figure 3b). The dramatic resistance increase of the unmodified NiO_*x*_ after the Ar plasma treatment via RF sputtering indicates a decrease in O_i_^″^ concentration in the NiO_*x*_ film, which reduces the NiO_*x*_ surface. This interpretation fits the results from XPS, which shows that Ar^+^ etching leads to a decrease in the Ni^3+^/Ni^2+^ ratio. The likely cause is that RF Ar plasma sputtering, similar to Ar^+^ etching, removes more of the lighter oxygen atoms than of the heavier Ni atoms, leading to V_O_^··^ and reduction in the NiO_*x*_ p-doping levels. The result is that the NiO_*x*_ is less p-type and becomes less conductive. The Ni_*y*_N layer modification decreases the resistance by around 20×, and while it increases after Ar plasma, the resistance is still six times smaller than that of NiO_*x*_ before Ar plasma treatment. The XPS measurements suggest an explanation for preserving the low resistance after Ar plasma by applying the Ni_*y*_N layer, namely, that the original Ni^3+^ concentration is retained. The results from UPS are consistent with the resistance decrease after Ni_*y*_N modification of NiO_*x*_. The energy of the VBM is closer to *E*_F_ after Ni_*y*_N modification; the IE, 5.1 eV, is smaller than that of the unmodified NiO_*x*_, 5.24 eV, which improves the contact with a gold probe (WF ∼5.1 eV). The VBM shift toward *E*_F_ as Ni_*y*_N gets thicker may explain the minor resistance increase after Ar plasma, as part of the Ni_*y*_N layer is etched away.

**Figure 4:**
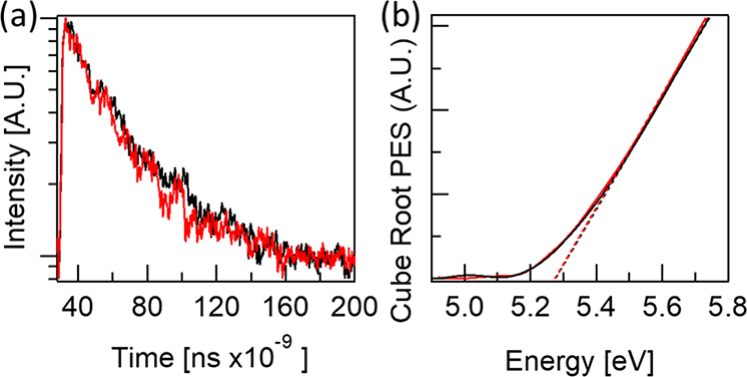
(a) TRPL and (b) PEYS measurements of MAPbI_3_, deposited on plasma-etched Ni_*y*_N-modified NiO_*x*_ (red) and unmodified NiO_*x*_ (black), both on an FTO substrate and after plasma etching.

After measuring the transparency and resistance of NiO_*x*_ with and without Ni_*y*_N, before and after plasma etching, we marked Ni_*y*_N-modified NiO_*x*_ after plasma treatment as the best candidate for PSCs. Thus, we deposited MAPbI_3_ on top of NiO_*x*_ with and without a Ni_*y*_N layer after plasma treatment and then performed time-resolved photoluminescence (TRPL) to measure differences in charge extraction after Ni_*y*_N deposition and plasma treatment. A fit of the PL decay to a double exponential curve typically indicates two kinds of decay mechanisms, which are common when HaP is deposited on a selective contact.^30−34^ A short lifetime τ_1_ corresponds to charge transfer and detrapping of charge due to light exposure, and a longer lifetime τ_2_ corresponds to recombinations at the interface and within the absorber. Because τ_1_ and τ_2_ were hard to distinguish, we chose to calculate only τ_2_ according to a monoexponential fit at a decay time > 60 ns.^35^ Similar lifetimes, 31 and 29 ns for MAPbI_3_ on NiO_*x*_ and Ni_*y*_N-modified NiO_*x*_, respectively (Figure 4a), indicate that Ni_*y*_N modification did not cause more (or less) recombination at the interface. Furthermore, PEYS, AFM, SEM, and XRD measurements (Figure 4b and Figures S5–S7) do not differ between MAPbI_3_ layers deposited on Ni_*y*_N-modified or unmodified NiO_*x*_.

AFM imaging of the surfaces before and after Ni_*y*_N modification does not show any difference in morphology (Figure S5).

SEM and XRD (Figures S6 and S7) measurements show no significant difference in MAPbI_3_ morphology and crystallinity (peak presence, positions, and widths). Both XRD analysis and SEM micrographs did not show any signs of PbI_2_ beyond the peak-to-noise ratio level.

PEYS (Figure 4b) yielded the same values for the MAPbI_3_ IE between samples deposited on NiO_*x*_ or Ni_*y*_N-modified NiO_*x*_ in dry N_2_.

The MAPbI_3_ surface morphology similarities before and after Ni_*y*_N modification of NiO_*x*_ and the PEYS and XRD results indicate and even imply that the solar cell efficiency improvement in these samples is caused by the Ni_*y*_N layer effect on the NiO_*x*_ selective contact rather than an effect on the MAPbI_3_ layer.

**Figure 5:**
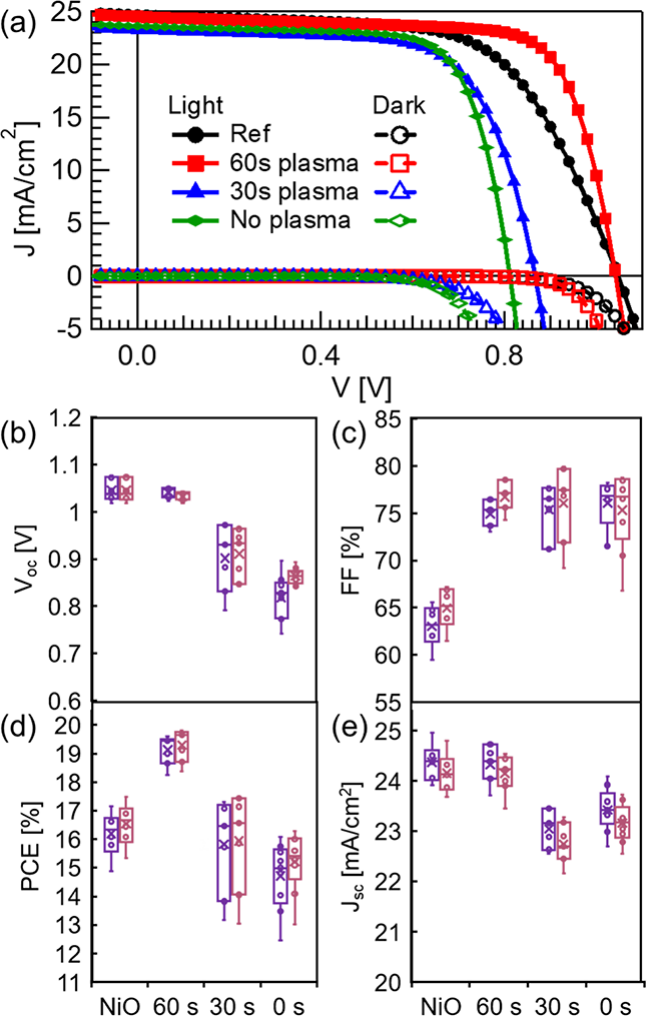
(a) *I*–*V* curves in the dark (dashed) and under illumination (solid) of representative PSCs with NiO_*x*_ (black circles) and an additional Ni_*y*_N layer after 60 s (red squares), 30 s (blue triangles), and no Ar plasma etching (green rhombohedrons). (b–e) Statistical distributions of the photovoltaic parameters for 30 PSCs employing as HTM NiO_*x*_ and NiO_*x*_ after depositing a Ni_*y*_N layer onto it, after 60 s, 30 s, and no Ar plasma etching, in the descending (pink) and ascending (purple) scans: (b) *V*_oc_, (c) FF, (d) PCE, and (e) *J*_sc_.

To test the photovoltaic activity of the Ni_*y*_N-modified cells, *I*–*V* measurements, in the dark and under illumination, were recorded on all complete PSCs in both ascending and descending voltage scan directions (Figure S8), but only the descending direction is shown for simplicity (Figure 5a). Statistics on 30 PSCs reveal that the Ni_*y*_N passivation layer affects the entire device in two main ways. As the etching time is decreased, the Ni_*y*_N layer becomes thicker, and the *V*_oc_ decreases from an average of 1.05 V for cells with unmodified NiO_*x*_ to 0.8 V for Ni_*y*_N with no plasma etching (Figure 5b). *V*_oc_ highly depends on the Ni_*y*_N thickness, and most of the 0.25 V *V*_oc_ loss is gained back as the Ar plasma treatment increases, and Ni_*y*_N gets thinner, up to a negligible value of 0.01 V. This effect of the Ni_*y*_N thickness on *V*_oc_ can be linked to the UPS-measured VBM shift of up to 0.37 eV toward *E*_F_ as the ∼2 nm Ni_*y*_N thickness increases, all the way to the Fermi level when the Ni_*y*_N is 40 nm thick (Figure S3). The similarity in *V*_oc_ between these two samples is consistent with the similar results obtained by TRPL. At the same time, the FF improves dramatically from a 65% average with unmodified NiO_*x*_ to 75% with the Ni_*y*_N layer, regardless of its thickness or the etching time (Figure 5c). The FF improvement for the Ni_*y*_N-modified PSCs indicates that despite its metallic properties, the Ni_*y*_N layer was thin enough so that no obvious damaging trap states formed at the interface with MAPbI_3_.

The minor decrease in *V*_oc_ and the significant improvement in the FF yield an overall PCE improvement, from a 16.5% average and a 17.4% record cell for the reference NiO_*x*_ to a 19.2% average and a 19.8% record cell for NiO_*x*_ with a Ni_*y*_N layer after 60 s of Ar plasma (Figure 5d). A slight decrease of 2 mA in *J*_sc_ for PSCs with a shorter Ni_*y*_N etching time (Figure 5e) is consistent with a slightly lower external quantum efficiency (EQE) (Figure S9) for these samples and is explained by the decrease in transparency, as seen in Figure 3a. Therefore, the proposed optimum use of our Ni_*y*_N modification is to have as thin a Ni_*y*_N layer on NiO_*x*_ as possible, which can passivate the HTL with a minimum loss in *V*_oc_ and transparency.

**Figure 6:**
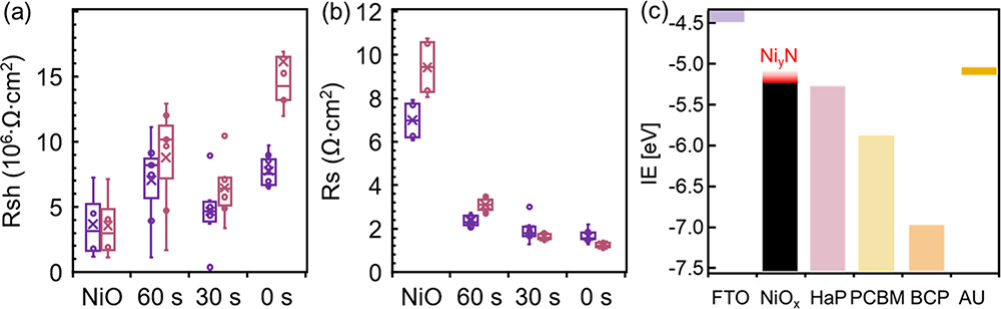
(a) Shunt resistance and (b) series resistance as measured from dark *I*–*V* measurements. (c) IE values (i.e., VBM), measured from the vacuum level, of the layers composing the SC stacks.

To further investigate the huge effect that the Ni_*y*_N layer has on the complete solar cell FF, we performed dark *I*–*V* measurements to determine the shunt resistance (*R*_sh_) and the series resistance (*R*_s_) of the SC (Figure 6a,b, respectively). *R*_sh_ increased with the Ni_*y*_N layer thickness, presumably due to the added coverage by the amorphous Ni_*y*_N layer, which inhibited electrical shorts between the layers. *R*_s_ dramatically dropped whenever Ni_*y*_N was deposited and decreased further with thicker Ni_*y*_N layers but with a minor trend. After summing up our former observations of UPS, XPS, transparency, and resistance, we conclude that the significant *R*_s_ decrease after Ni_*y*_N deposition is due to a higher Ni^3+^ concentration within NiO_*x*_, when the Ni_*y*_N layer is present. The minor trend of *R*_s_ decrease is inversely related to the Ar plasma etching time and follows the thickness of the Ni_*y*_N layer. Based on the interpretation of the UPS measurement (Figure 2a and Figure S3), we ascribe this to a VBM shift toward the vacuum level that increases the driving force for hole extraction from the HaP film but at the expense of the *V*_oc_ (Figure 6c).

**Figure 7:**
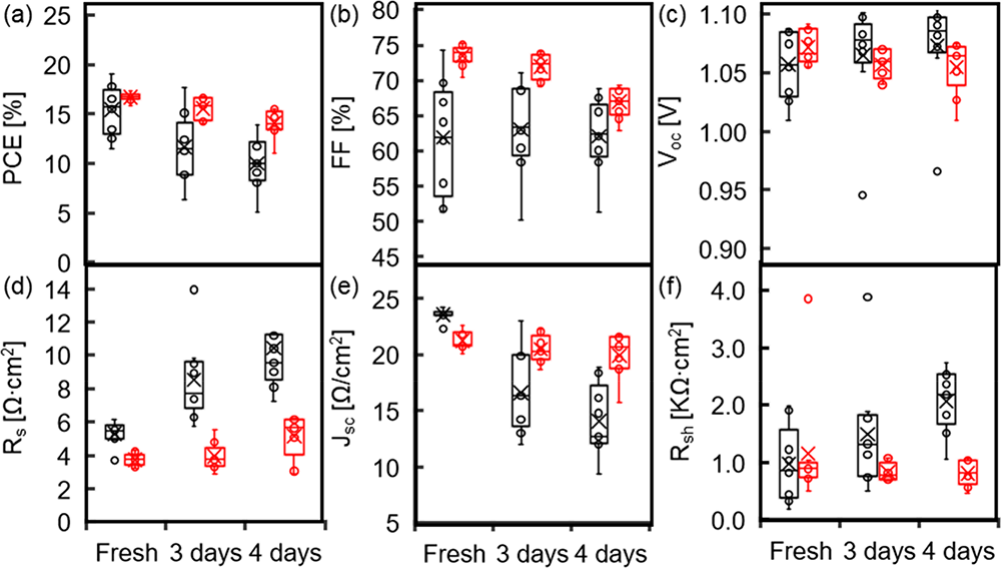
Statistical photovoltaic parameters of 18 PSCs over 4 days, employing NiO_*x*_ as an HTL with Ni_*y*_N (red) and without (black) modification. Measurements were done in a descending scan direction: (a) PCE, (b) FF, (c) *V*_oc_, (d) *R*_s_, (e) *J*_sc_, and (g) *R*_sh_.

To test the effect of the Ni_*y*_N modification on cell stability, 18 cells were measured over 4 days at room temperature in a N_2_-filled chamber with a controlled environment and relative humidity that did not surpass 5%. The cells were held in the dark between the measurements and were exposed to light for 4 h before and naturally during measurements. For both types of cells, the PCE decreased over the 4 days. However, on average, the reference cell PCE values were reduced by almost 50%, from 15.9 to 9%, while those of the Ni_*y*_N-modified cells were reduced only by 15%, from 16.5 to 14% (Figure 7a).

Further investigation of the SC statistics reveals a different aging mechanism for cells with Ni_*y*_N than those without it. The *V*_oc_ and *J*_sc_ of both types of cells show inverse changes over the 4 days of testing. The *J*_sc_ of the reference cells decreased by 40% to, on average, 14 mA/cm^2^, whereas the *J*_sc_ of the Ni_*y*_N-modified cells remained almost constant at 20 mA/cm^2^ (Figure 7e). The *V*_oc_ of the reference cells increased by 3% up to 1.1 V, and the *V*_oc_ of the Ni_*y*_N-modified cells decreased by 8% down to 0.95 V (Figure 7c). The FF values remained the same (63% without and 73% with Ni_*y*_N modified-NiO_*x*_; see Figure 7b). The series and shunt resistances of the reference cells increased gradually over 4 days by more than 90%. However, the series and the shunt resistances of the Ni_*y*_N-modified cells remained constant for 3 days, and only on the fourth day did the series resistance increase by 30% (Figure 7d,f), still a much lower factor than that of the reference cells. The rapid *J*_sc_ reduction and the *R*_s_ increase in untreated PSCs are prominent evidence of a reaction at the NiO_*x*_–MAPbI_3_ interface inhibited by the Ni_*y*_N treatment. The Ni_*y*_N layer plays a dual role, as it helps maintain the Ni^3+^ concentration, passivates the NiO_*x*_–MAPbI_3_ interface, and presumably prevents a reaction between the more active Ni^3+^ and MAPbI_3_. These two features of the Ni_*y*_N layer improve the solar device FF and, most importantly, cell stability.

**Figure 8:**
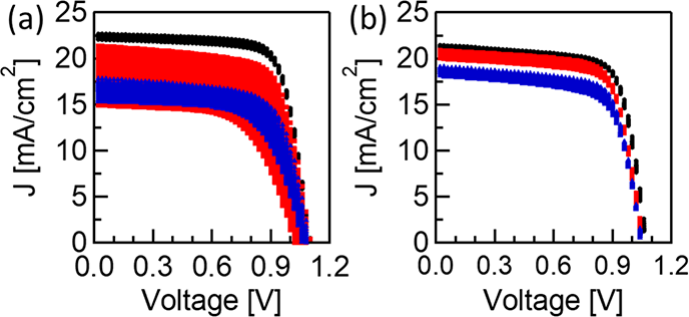
*I*–*V* curves of fresh (black), 3-day-old (red), and 4-day-old (blue) cells with (a) a NiO_*x*_ HTL and (b) a Ni_*y*_N-modified HTL.

To further investigate the current reduction source, we measured *I*–*V* characteristics over 4 days, but this time we repeated the *I*–*V* measurements for 100 cycles every day (Figure 8a,b). The reference cells exhibit an obvious current reduction when the number of cycles increases, while the Ni_*y*_N-modified cells do not. Although in the initial response, the shunt resistance of the reference cell is higher than that of the Ni_*y*_N-modified SC, its series resistance is higher by 150%, which leads to the two types of cells having equal FFs. After 4 days of cycling, the series resistance of the reference cell increased by 70%. Similar to what Khenkin et al.^36^ showed, the initial *V*_oc_ of the reference PSC started every day at 1.1 V but decreased as the number of cycles progressed, which is not the case for the Ni_*y*_N-modified cells. We interpret this finding as an acceleration of the reaction at the interface by the constant change in the electric field applied to the SC under working conditions. The *J*_sc_ and *R*_s_ reductions suggest that an insulating interfacial layer formed between NiO_*x*_ and MAPbI_3_. In contrast, in the cells prepared with Ni_*y*_N-treated NiO_*x*_, *J*_sc_ only undergoes a 3 mA/cm^2^ loss over the 100 cycles. The constant *R*_s_ and *R*_sh_ over time indicate no change in charge transport and therefore no change of the material under working conditions. These results altogether support that Ni_*y*_N modification inhibits reaction at the NiO_*x*_–MAPbI_3_ interface and prevents the formation of a blocking layer that deteriorates the SC performance.

## Conclusions

We used a physical vapor deposition method to improve NiO_*x*_ HTLs with Ni_*y*_N inorganic layers, which is the first time that such a modification was ever done. We showed that Ni_*y*_N plays a dual role, improving the PSC’s efficiency and stabilizing the NiO_*x*_–HaP interface. The Ni_*y*_N layer formed on NiO_*x*_ retains the Ni^3+^ species within the nickel oxide bulk, preserving its conductivity and thereby improving the overall cell efficiency. Furthermore, the same Ni_*y*_N layer protects HaP from the reactive Ni^3+^ species, necessary for nickel oxide conductivity, and prevents degradation of HaP, giving devices superior stability over those built with untreated reference NiO films. Although, with time, some of the Ni_*y*_N might be oxidized by the NiO_*x*_ layer, in a well-encapsulated device, the interfacial reactivity can be kept below the level where charged defects will start to change the overall defect density of the transport and absorber layers. These interactions should be investigated further beyond the scope of this study. We also showed that when Ni_*y*_N is thin enough, its small energy gap, which can block sunlight and introduce traps for charge carriers at the interface, has a negligible effect on the overall SC efficiency. Our first reported solid-state, inorganic, in situ passivation route via RF sputtering deposition presents a major step toward solvent-free fabrication of reproducible and stable PSCs.

## Supporting Information Available

Scheme of the cell structure, XPS peak fitting of the O 1s peak, UPS and IPES measurements of ∼40 nm Ni_*y*_N, energy diagrams for the NiO_*x*_ and Ni_*y*_N-modified NiO_*x*_ vs *E*_F_, AFM scans, SEM micrographs of MAPbI_3_, XRD plot for MAPbI_3_, *I*–*V* curves in ascending and descending directions, and EQE of PSCs with different Ni_*y*_N etching times (PDF)
