# Supplementary material for: NiN-Passivated NiO Hole-Transport Layer Improves Halide Perovskite-Based Solar Cell
Source: ACS Appl Mater Interfaces. 2022 Oct 13;14(42):47587–94. doi: 10.1021/acsami.2c11701 (PMC9614719; doi:10.1021/acsami.2c11701)
Supplement: Supplementary file 1 — am2c11701_si_001.pdf [file am2c11701_si_001.pdf]

Supporting information

for

NiN-passivated NiO hole-transport layer improves halide perovskite-based solar cell

Anat Itzhak,[1‡] Xu He,[2‡] Adi Kama,[1] Sujit Kumar,[1, 3] Michal Ejgenberg,[1] Antoine Kahn,[2] David Cahen[1, 3*]*

[1] Department of Chemistry and Bar-Ilan Institute for Nanotechnology & Advanced Materials, Bar-Ilan University, Ramat Gan 5290002, Israel

[2] Department of Electrical and Computer Engineering, Princeton University, Princeton, NJ 08544, USA

[3] Weizmann Institute of Science, Rehovot 7610001, Israel

E-mail: david.cahen@weizmann.ac.il

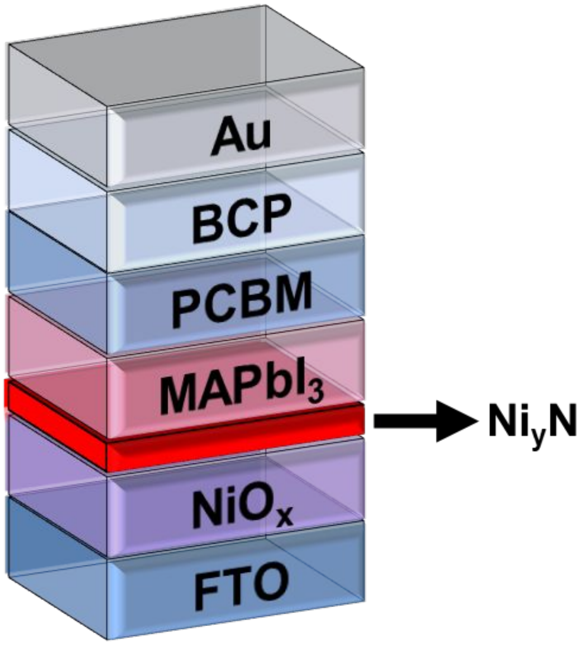

Figure S1. A scheme of the complete solar cell structure, the scheme is not to scale.

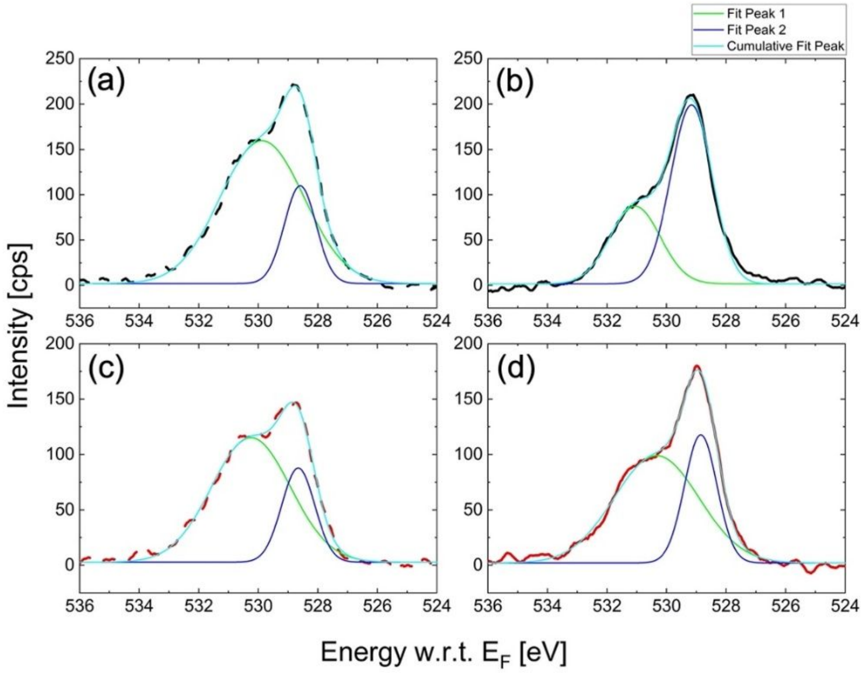

Figure S2. Peak fitting of O 1s peak for unmodified NiO_x (black) (a) before Ar^+ etching (dashed) (b) after Ar^+ etching (solid) and Ni_yN-modified NiO_x (red) (c) before Ar^+ etching (dashed) (d) after Ar^+ etching (solid). The green curve assigned to oxygen bound to a Ni^{3+}, and the purple curve assigned to oxygen bound to a Ni^{2+}.

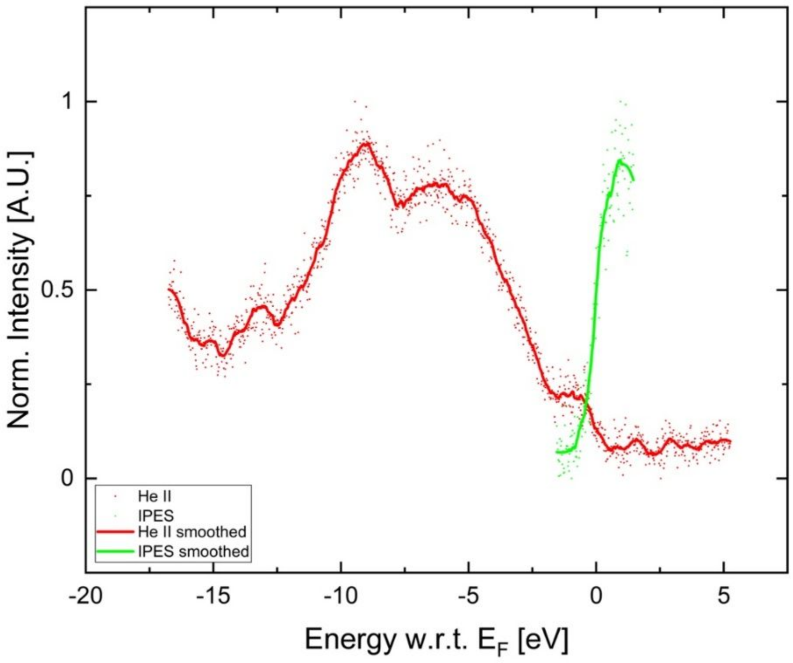

Figure S3. UPS (red) and inverse photoemission spectroscopy ,IPES, (green) measurements of ~40nm Ni_yN.

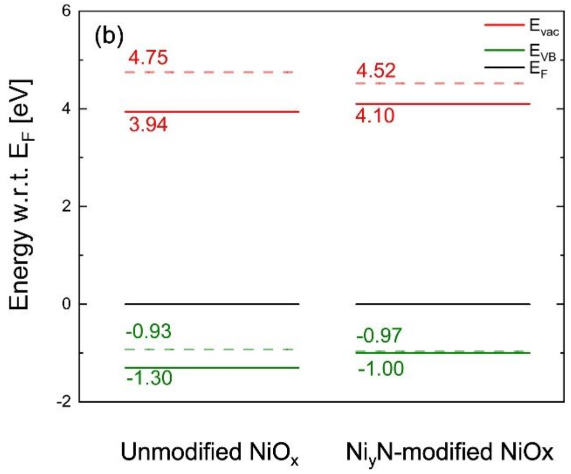

Figure S4. Energy diagrams for the NiO_x (left) and Ni_yN-modified NiO_x (right) before (dashed) and after (solid) 5s Ar^+ etching. All energy levels are plotted with respect to the Fermi level (E_f).

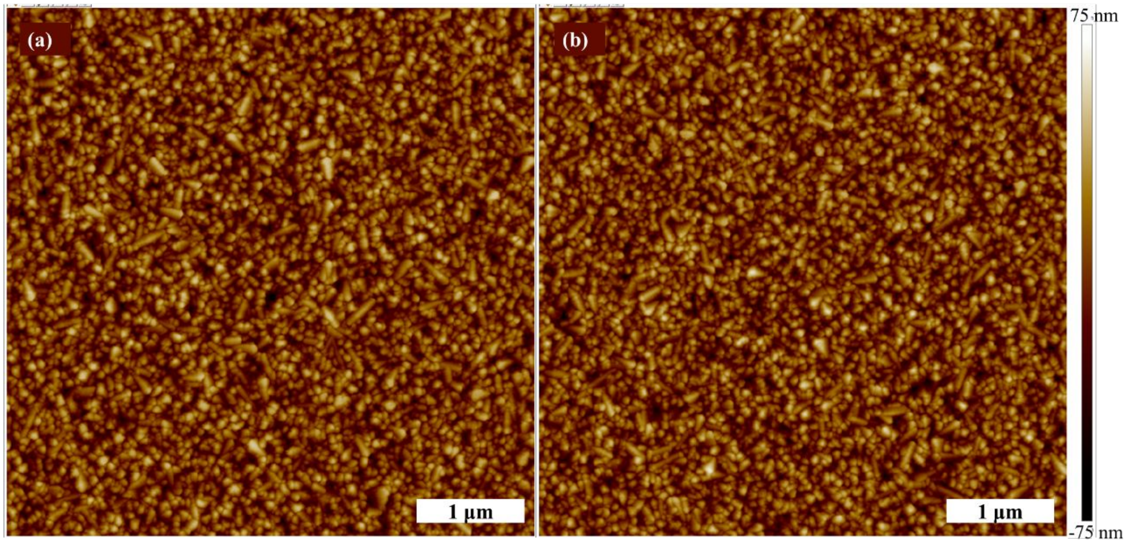

Figure S5. AFM scans of a) Ni_yN-modified and b) unmodified NiO_x sample, after plasma etching.

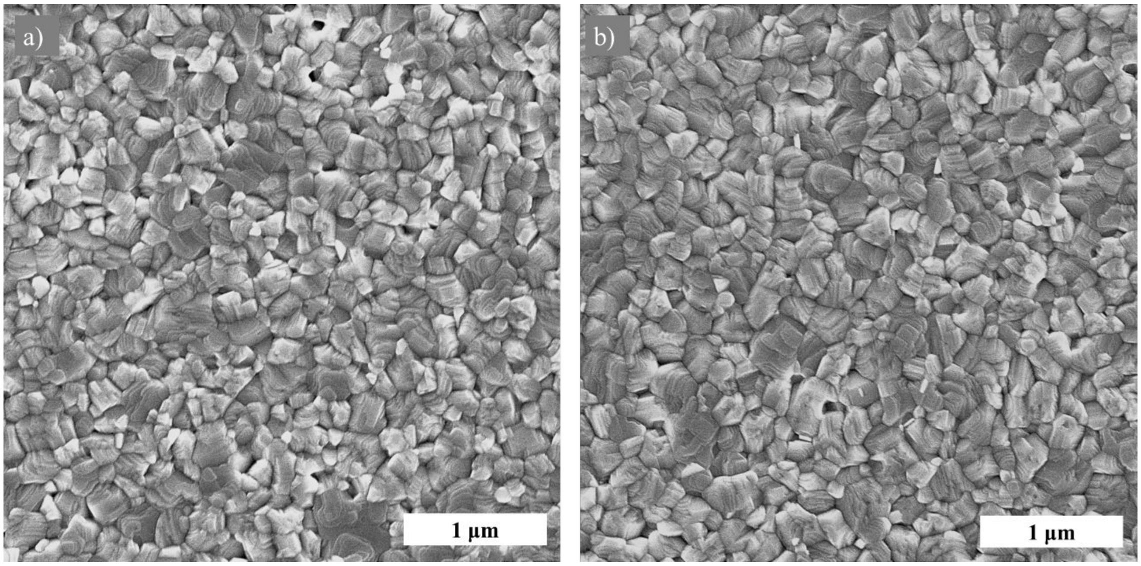

Figure S6. SEM micrographs of $MAPbI_3$, deposited on plasma etched a) Ni_yN-modified and b) unmodified NiO_x films.

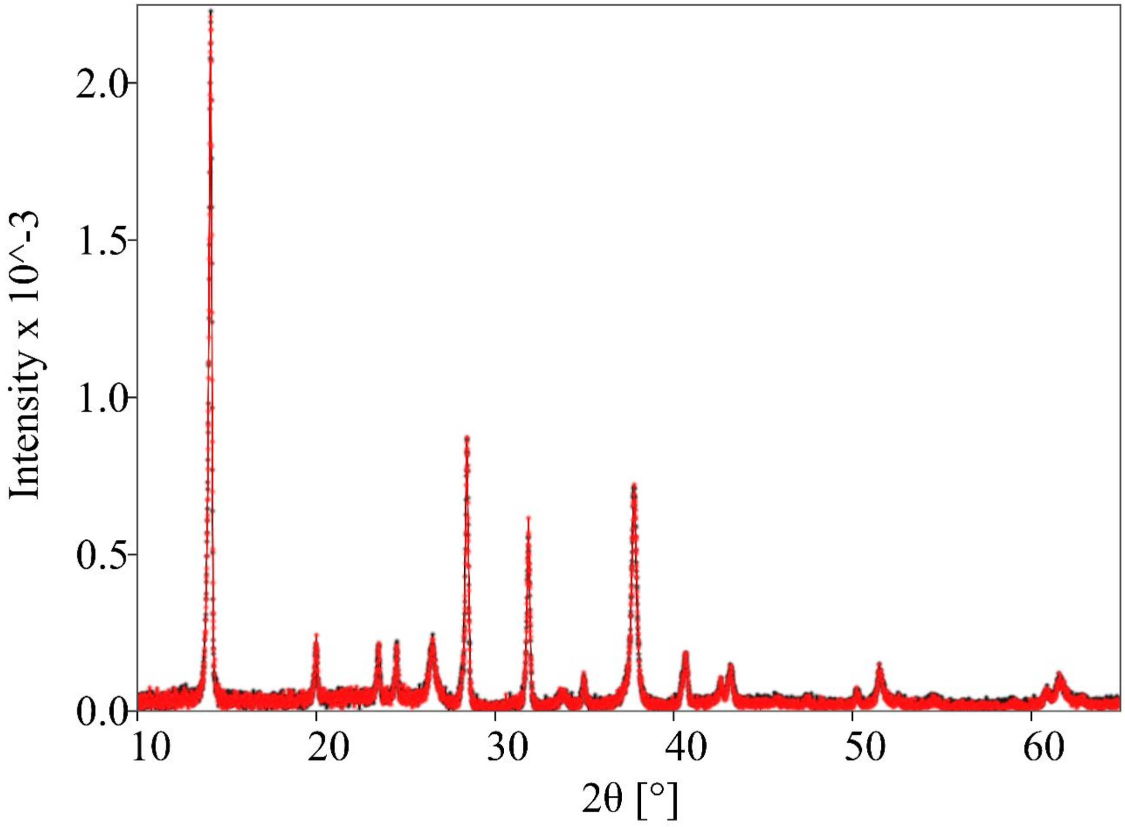

Figure S7. XRD plot for $MAPbI_3$ films that were deposited on plasma etched Ni_yN-modified (red) and unmodified NiO_x (black). Note that the plots overlap.

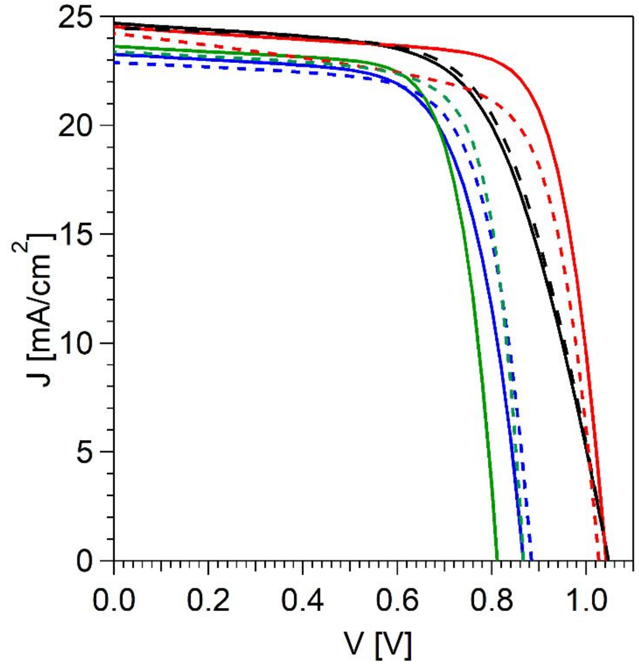

Figure S8. I-V curves in ascending and descending directions of representative solar cells with NiO_x (black) and additional Ni_yN layer after 60 s (red), 30 s (blue), and no Ar plasma etching (green).

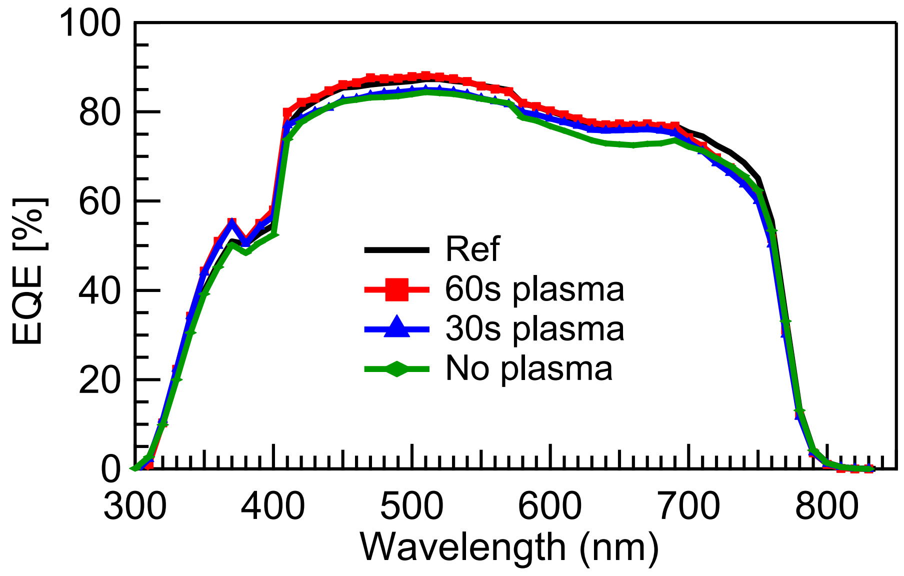

Figure S9. External quantum efficiency, EQE (a.k.a. Incident Photon to Current Conversion Efficiency, IPCE) curves of representative PSCs with NiO_x (black circles) and with NiO_x plus additional Ni_yN layer after 60 s (red squares), 30 s (blue triangles), and no Ar plasma etching (green rhombohedrons).
